# The uses and implications of avian vocalizations for conservation planning

**DOI:** 10.1111/cobi.13465

**Published:** 2020-04-15

**Authors:** Rebecca N. Lewis, Leah J. Williams, R. Tucker Gilman

**Affiliations:** ^1^ Department of Earth and Environmental Sciences University of Manchester Manchester M13 9PL U.K.; ^2^ North of England Zoological Society (Chester Zoo) Chester CH2 1LH U.K.

**Keywords:** behavior, bioacoustics, birdsong, conservation interventions, cultural evolution, monitoring, bioacústica, canto de aves, comportamiento, evolución cultural, intervenciones de conservación, monitoreo, 行为, 保护干预措施, 鸟鸣, 生物声学, 监测, 文化演化

## Abstract

There is a growing recognition that animal behavior can affect wildlife conservation, but there have been few direct studies of animal behavior in conservation programs. However, a great deal of existing behavioral research can be applied in the context of conservation. Research on avian vocalizations provides an excellent example. The conspicuous nature of the vocal behavior of birds makes it a useful tool for monitoring populations and measuring biodiversity, but the importance of vocalizations in conservation goes beyond monitoring. Geographic song variants with population‐specific signatures, or dialects, can affect territory formation and mate choice. Dialects are influenced by cultural evolution and natural selection and changes can accumulate even during the timescale of conservation interventions, such as translocations, reintroductions, and ex situ breeding. Information from existing research into avian vocalizations can be used to improve conservation planning and increase the success of interventions. Vocalizations can confer a number of benefits for conservation practitioners through monitoring, providing baseline data on populations and individuals. However, the influence of cultural variation on territory formation, mate choice, and gene flow should be taken into account because cultural differences could create obstacles for conservation programs that bring birds from multiple populations together and so reduce the success of interventions.

## Abstract 2

*Article impact statement*: Avian vocalizations are well studied but should be considered more both as a tool and potential hindrance in conservation interventions.

## Abstract (es)

Implicaciones y Usos de las Vocalizaciones de Aves para la Conservación de la Planeación

**Resumen**

Hay un creciente reconocimiento de que el comportamiento animal puede afectar a la conservación de la fauna, pero ha habido pocos estudios directos del comportamiento animal en los programas de conservación. Sin embargo, una gran cantidad de la investigación existente sobre el comportamiento puede aplicarse en el contexto de la conservación. Las investigaciones sobre las vocalizaciones de aves son un excelente ejemplo. La naturaleza conspicua del comportamiento vocal de las aves hace que sea una herramienta útil para el monitoreo de las poblaciones y las medidas de la biodiversidad, pero la importancia de las vocalizaciones en la conservación va más allá del monitoreo. Las variantes geográficas de canto con firmas específicas para cada población, también conocidas como dialectos, pueden afectar a la formación del territorio y a la elección de pareja. Los dialectos están influenciados por la evolución cultural y la selección natural y los cambios pueden acumularse incluso durante la escala de tiempo de las intervenciones de conservación, como las reubicaciones, las reintroducciones y la reproducción ex situ. La información disponible a partir de las investigaciones existentes sobre las vocalizaciones de aves puede usarse para mejorar la planeación de la conservación e incrementar el éxito de las intervenciones. Las vocalizaciones pueden otorgar un número de beneficios para quienes practican la conservación a través del monitoreo, proporcionando información de la línea base de las poblaciones y los individuos. Sin embargo, la influencia de la variación cultural sobre la formación del territorio, la elección de pareja y el flujo génico deberían considerarse ya que las diferencias culturales podrían crear obstáculos para los programas de conservación que agrupan a aves de diferentes poblaciones y con ello reducen el éxito de las intervenciones.

## 摘要

目前, 人们已越来越多地认识到动物行为可以影响野生动物保护, 但在保护项目中对动物行为的直接研究还很少。不过, 大量现有的行为研究都可以应用于到保护之中。鸟类声音的研究就是一个很好的例子。鸟类鸣叫行为十分引人注意, 可以作为监测种群和衡量生物多样性的有用工具, 但鸟鸣在保护中的重要意义不仅仅在于监测。鸟类的叫声具有特定种群特征的地理变异, 或称之为方言, 这可以影响领域形成和配偶选择。鸟类的方言受文化演化和自然选择的影响, 且在物种保护措施 (如异地放归、重引入、迁地繁殖) 的时间尺度内也会不断积累变化。来自现有鸟鸣研究的信息可用于改进保护计划、促进干预措施的成功。鸟鸣研究可以通过监测, 提供种群和个体的基础数据, 为保护实践者带来许多好处。然而, 保护实践者还应该考虑鸟类种群的文化差异对领域形成、配偶选择和基因流的影响, 因为文化差异可能会阻碍将来自多个种群的鸟类聚集在一起的保护计划的实施, 从而降低干预措施的成功率。**【翻译: 胡怡思; 审校: 聂永刚】**

## Introduction

A recent assessment by BirdLife International (2018) showed that around 13% of all extant bird species are globally threatened and many more are in decline. Many conservation measures have been attempted to help reverse declines; intensive interventions, such as ex situ breeding, reintroductions, and translocations, have proven valuable (e.g., Cade & Jones 1993; Miskelly & Powlesland 2013). When planning these types of interventions, it is important to consider how animal behaviors may affect success.

Although attention to animal behavior in conservation has increased in recent years, there is still a broad consensus that information on animal behavior is underutilized (Angeloni et al. 2008; Berger‐Tal et al. 2016). Thus, future research should examine behavior and conservation in tandem. However, there is already considerable behavioral research available that has implications for conservation and can be used to guide management strategies. Vocal communication is an easily detected, conspicuous behavior and is well described for many species (Lovette & Fitzpatrick 2016). We summarized the relevant background information on variation in avian vocalizations, considered how vocalizations influence key processes of conservation interest, and explored the potential roles of vocalizations in conservation applications.

## Variation in Avian Vocalizations

Avian vocalizations can vary in a number of ways based on temporal factors (Table 1), spectral characters, and the elements, syllables, and phrases vocalizations are composed of. Variation among species, often used for conspecific recognition, and the role of among‐species variation in territory defense (e.g., North Island Saddlebacks [*Philesturnus rufusater*] [Parker et al. 2010]) and mate choice (e.g., Medium Ground Finches [*Geospiza fortis*] and Cactus Finches [*G. scandens*] [Grant & Grant 1996]) are well understood (Catchpole & Slater 2008). However, variation can occur at smaller scales within and among populations.

**Table 1:** Glossary of specialist terms pertaining to bioacoustics and song learning.*

Term	Definition
Amplitude	volume of sound, measured as height of sound waves in vocalization
Call	short, simple vocalizations used for social cohesion, parent–offspring communication, aggression, and signaling danger
Closed‐ended learner	bird in which song learning is restricted to a short period, usually the first year of life (Beecher & Brenowitz 2005)
Cultural variation	variation among populations in information or behaviors shared by individuals and acquired from conspecifics by social learning (Whitehead & Rendell 2015)
Dialect	sets of geographic vocalization variants with distinct, population‐specific vocal features
Elements	smallest divisions of birdsong, also referred to as notes
Frequency	wavelength of sound; shorter wavelengths produce higher frequencies; frequency of a call can be measured as the maximum (highest frequency), minimum (lowest frequency), mean (across the song or individual elements), and peak (frequency with the highest amplitude)
Open‐ended learner	bird in which song learning can occur throughout life (Beecher & Brenowitz 2005)
Phrases	series of units (usually syllables) occurring together in a particular pattern
Repertoire	full set of vocalizations that a single individual produces
Repertoire size	total number of different vocalizations an individual produces, usually measured as the number of different song types
Song	loud, long, and usually complex vocalizations most often used in courtship and territory defense
Song complexity	variously, song repertoire size, note repertoire, versatility, nonlinearity, and standard deviation of frequencies have been proposed as definitions of complexity and all may act as honest signals of fitness (Soma et al. 2006; Pearse et al. 2018)
Song rate	number of songs produced by an individual per unit time
Spectral factors	factors relating to the frequency of vocalizations
Syllables	building blocks of phrases, can be complex (containing multiple elements) or simple (containing only 1 or 2 elements)
Temporal factors	factors relating to the timing of vocalizations
Withdrawal of learning	rapid song innovation following colonization by founders that dispersed before song crystallization (i.e., before they produce stereotyped, adult songs) (Thielcke 1973)

*Unless otherwise stated, definitions are based on Catchpole and Slater (2008) and Lovette and Fitzpatrick (2016).

Within populations, individual variation is common. Such variation may be affected by intrinsic factors, such as morphology: body size affects spectral characters and beak shape affects temporal factors (Derryberry et al. 2018; García & Tubaro 2018). Vocal repertoire and repertoire size can also vary widely between individuals of the same species (Krebs & Kroodsma 1980).

Among populations, cultural variation in vocalizations, known as dialect, has been reported in many species (e.g., Wright et al. 2008; Robin et al. 2011). There are a number of suggested mechanisms for dialect formation and evolution (Catchpole & Slater 2008), and understanding these is essential to predicting the possible impacts of dialects on conservation programs.

Cultural drift describes the random accumulation of song mutations due to copying errors and improvisation (Mundinger 1980). The action of drift is more severe in smaller populations, especially in fragmented environments, which are commonplace in conservation (Laiolo & Tella 2007). Over 19 years, calls of the Yellow‐naped Amazon (*Amazona auropalliata*) showed a lower degree of stability in the smaller northern population relative to the larger southern population (Wright et al. 2008). The rate of drift may also be influenced by song traits. In White‐bellied Shortwings (*Sholicola major*), simple songs are still similar in populations that have been separate for thousands of years, but differences in complex song clusters are apparent in populations separated for a comparably shorter time (100–150 years) (Purushotham & Robin 2016).

Cultural traits like song diversity can be lost during population bottlenecks, such as the colonization of new habitats (e.g., Baker 1996) or population reductions following habitat loss or fragmentation (Laiolo & Tella 2007). Hill et al. (2013) reported reduced syllable diversity and a lower percentage of trills in the threatened Chatham Island Tui (*Prosthemadera novaeseelandiae chathamensis*) relative to its mainland counterpart (*P. n. novaeseelandiae*). However, it is often impossible to detect bottleneck events from song diversity due to the continued action of drift or withdrawal of learning (Potvin & Clegg 2015). In such cases, past bottlenecks may be evidenced by low shared syllables between populations (e.g., Lang & Barlow 1997).

The acoustic adaptation hypothesis predicts that habitat‐dependent selection shapes song evolution. Different habitats have different sound transmission properties: complex vegetation causes greater attenuation, particularly of high‐frequency sounds, than more open habitats (Brumm & Naguib 2009). A meta‐analysis of studies examining acoustic adaptation showed a small overall effect of habitat on frequency across oscine and suboscine species; lower frequencies (minimum, maximum, and peak) and smaller frequency bandwidths occur in closed habitats (Boncoraglio & Saino 2007).

Biotic noise is highly variable and may be hard for birds to avoid. There are increasing examples of spectral and temporal partitioning in the acoustic signals of wild birds, both in response to other birds (Ficken et al. 1974; Planque & Slabbekoorn 2007; Luther 2009) and other taxa (Sueur 2002; Hart et al. 2015). Grant and Grant (2010) detail song changes of *G. fortis* and *G. scandens* after the arrival of *G. magnirostris* on Daphne Major in 1983. Over the study period (1983–2010), the song traits of *G. fortis* and *G. scandens* dispersed away from those of *G. magnirostris*; changes included shorter songs and increased trill rate, which could not be explained by other changes in the environment.

It is likely that dialect formation is influenced by a combination of the mechanisms mentioned above. Given the number of possible influences, it is difficult to disentangle the driving factors, and factors may change in importance over time (Potvin & Clegg 2015; Purushotham & Robin 2016).

## Importance of Variation in Vocalizations to Processes of Conservation Interest

Variation in vocalizations among populations can play an important role in a number of processes important for population persistence, such as territory maintenance, mate choice, and gene flow.

### Territory Formation and Defense

In many species, holding territories is essential for resource acquisition and the formation and maintenance of pair bonds and so improves mating success (Hinde 1956). Successful territory maintenance relies on the ability to identify conspecifics and respond by defending the territory. However, birds respond more strongly to unfamiliar songs of their own dialect and to songs that are more similar to their own than to foreign dialects (e.g., Searcy 1997). As a result, birds may fail to adequately defend their territories from conspecifics with foreign dialects. Irwin et al. (2001) examined responses of the Greenish Warbler (*Phylloscopus trochiloides*), a species where songs vary clinally in a ring. Along the cline, males respond to playback from recordings taken 1000–1500 km away but no further. When the 2 terminal subspecies, which have come into secondary contact and exhibit large differences in dialect, were tested, neither responded to the other regardless of distance between the male and the recording. The combined evidence from territory studies suggests birds may find it difficult to defend territories where foreign dialects are prevalent (Slabbekoorn & Smith 2002), which could reduce access to food, nest sites, and mates. This may cause problems if birds with different dialects are brought together during conservation interventions (Parker et al. 2010; Bradley et al. 2013; Valderrama et al. 2013).

### Mate Choice

Birdsong plays an important role in female mate choice. There is evidence that performance‐related factors, such as amplitude and rate (e.g., Ballentine et al. 2004 but see Kroodsma 2017 for critique), song complexity, and repertoire size (Searcy 1992; Byers & Kroodsma 2009) may influence female preference and could act as honest signals of male quality (Gil & Gahr 2002). Dialect can also influence female mate choice. Female preference for local dialects can promote assortative mating, where animals select mates genetically or phenotypically similar to themselves (Jiang et al. 2013). Searcy et al. (2002) found that female Song Sparrows (*Melospiza melodia*) showed similar responses to local and nearby (18 km) foreign dialects, but discriminated against dialects from greater distances (34, 68, and 135 km).

Selectiveness in mating can lead to an increased probability of mate rejection, reducing overall mating rates. Where mating opportunities are limited, as in some small, endangered populations, this may result in fewer individuals finding mates and breeding, potentially contributing to population declines and extinction (Bessa‐Gomes et al. 2003). Assortative mating plays a key role in premating reproductive isolation, possibly resulting in speciation (Kirkpatrick 2000; Verzijden et al. 2012), and can promote reproductive isolation at secondary contact (Grant & Grant 2002)—including during conservation interventions when previously separated populations are brought together. This has been reported following multiple conservation interventions in the North Island Kokako (*Callaeas wilsoni*) (Bradley et al. 2014).

Female preference for local dialects could be adaptive; females may gain fitness advantages by choosing males from their natal region (Slabbekoorn & Smith 2002; Podos & Warren 2007). Although singing a local dialect should not be inherently more costly than singing a foreign dialect (Nowicki & Searcy 2005), dialects could act as behavioral markers for other traits. In the Red Crossbill (*Loxia curvirostra*), song and bill morphology are strongly correlated. Mating within the local population, signaled by dialect, prevents the production of offspring with intermediate, less fit phenotypes (Snowberg & Benkman 2007). Local dialect may also signal males with local experience (Searcy 1982) who may be better able to secure resources for females and their offspring, thus providing direct benefits to choosy females.

Dialect preferences could also be a nonadaptive result of familiarity (Slabbekoorn & Smith 2002). Female White‐crowned Sparrows (*Zonotrichia leucophrys*) in mixed‐dialect populations show neither preference for their fathers’ dialects nor consistent preference for any dialect across successive breeding seasons (Chilton et al. 1990). In captivity, females do not respond differently to 2 dialects they commonly hear within their population, but show reduced responses to a dialect from a different population (Chilton et al. 1996). These results suggest that females from mixed‐dialect populations can distinguish between dialects, but show no difference in preference among songs they commonly hear. Females exhibiting a preference (for father's dialect or otherwise) would be expected to mate with birds of the same dialect across seasons. However, if preferences do not align with mate choice in the field, dialect may not have a profound effect on mating during conservation.

### Gene Flow

If dialects contribute to mate choice, they may reduce gene flow by reducing breeding between populations. Such inbreeding can affect fitness and affect both individual and population performance (Keller & Waller 2002). For example, inbreeding reduced hatching success, fledgling survival, and recruitment in the Red‐cockaded Woodpecker (*Picoides borealis*) (Daniels & Walters 2000).

A number of studies report substantial genetic mixing between populations despite dialect differences (e.g., Orange‐tufted Sunbirds [*Cinnyris bouvieri*] [Leader et al. 2008] and Puget Sound White‐crowned Sparrow [*Zonotrichia leucophrys pugetensis*] [Poesel et al. 2017]). Other studies show some genetic structuring related to dialect differences (e.g., Mountain White‐crowned Sparrows [*Zonotrichia leucophrys oriantha*] [MacDougall‐Shackleton & MacDougall‐Shackleton 2001]). Even small reductions in gene flow can reduce the effective population size, potentially contributing to reduced heterozygosity and Allee effects (Chesser et al. 1993).

Although dialects could limit gene flow between populations and increase genetic differentiation, other factors may also contribute. In particular, dialect differences are often related to distance between populations. In the White‐bellied Shortwing, song and genetic differences appear highly correlated. However, when controlling for geographic distance and dispersal barriers, spectral and syntax differences are not correlated with genetics (Purushotham & Robin 2016).

There is little consensus on the impact of dialects on gene flow, and some studies even show conflicting results for the same species and populations (e.g., Nuttall's White‐crowned Sparrow [*Zonotrichia leucophrys nuttali*] [Baker & Mewaldt 1978; Petrinovich et al. 1981; Baker et al. 1982; Zink & Barrowclough 1984; Hafner & Petersen 1985; Soha et al. 2004]). This is likely due to methodological differences (e.g., marker used, scale of study, and populations or subspecies chosen). The significance of dialect for gene flow is likely to vary by species due to life‐history traits (e.g., dispersal rates and mating systems) and vocal learning: birds learning song before dispersal are more likely to be affected than those learning throughout life (Podos & Warren 2007). Overall, there is little evidence to suggest that dialect alone could completely prevent gene flow between populations: even a few cross‐dialect pairs per generation would be sufficient to prevent divergence (Potvin et al. 2013). More information is required to provide useful evidence for conservation practitioners. Monitoring gene flow and dispersal events across a range of species, particularly endangered species during conservation interventions, should be a priority for researchers examining genetics in avian conservation.

## Uses and Implications of Avian Vocalizations for Conservation

Understanding the biology of avian vocalizations and their impact on population processes can help one appreciate the role of vocal behavior during conservation. We considered potential applications for the use of avian vocalizations (summarized in Table 2) and the potential negative effects of variation in vocalizations and how they might be overcome (summarized in Table 3).

**Table 2:** Summary of the uses of bioacoustics in conservation

Conservation activity	Use of bioacoustics	Possible taxa	Potential problem	Proposed solution	Reference
Collecting baseline data	detecting species presence or absence, identifying preferred habitats, etc.	species producing vocalizations	lack of standardized methods for acoustic detection surveys	produce standard protocols so studies are comparable	Teixeira et al. 2019
Conducting censuses	individual discrimination to improve resolution	all vocally active species (Terry et al. 2005)	requires all birds to vocalize during census period or for researchers to understand the proportion and demographics vocalizing	research species' vocalizations prior to census where possible	reviewed by Terry et al. 2005
Assessing life history traits/fitness	recognition of individual animals	all vocally active species (Terry et al. 2005)	assigning vocalizations to individuals can be challenging	investigate vocal individuality prior to study onset	reviewed by Terry et al. 2005
Assessing responses to perturbations	detecting presence or absence, changes in activity, differences in biodiversity	species producing vocalizations			e.g. Deichmann et al. 2017
Assessing success of conservation interventions	(as above)	species producing vocalizations			e.g. Buxton & Jones 2012; Metcalf et al. 2019
Attraction of single taxa to new habitats	playback of conspecifics to replicate conspecific attraction	e.g. territorial songbirds, colonial seabirds	unsuccessful attraction of target species	understanding of species’ biology and ecology – especially habitat, carrying capacity, and community structure	e.g. Reed & Dobson 1993; Ward & Schlossberg 2004; Hahn & Silverman 2007; Bayard & Elphick 2012
			potential declines of nontarget species (particular concern if multiple endangered taxa are present)		
Attraction of multiple taxa to new habitats	(as above)	(as above)	(as above)	(as above)	e.g. DeJong et al. 2015

**Table 3:** A summary of the implications of avian vocalizations in conservation

Conservation problem	Acoustic implications	Taxa to consider	Proposed solution	Reference
**In situ conservation**				
Habitat fragmentation	vocal divergence through differential drift and acoustic adaptation in separated populations	vocal learners – parrots, hummingbirds, songbirds, corvids (Catchpole & Slater 2008; Bluff et al. 2010)	conserve species as contiguous populations or maintain or improve connectivity	Laiolo & Tella 2005
Land use and habitat change	vocal change through acoustic adaptation vocalizations poorly adapted to transmission in the new environment	species producing vocalizations closed‐ended learners e.g. estrildid finches and sparrows (Brenowitz & Beecher 2005)	minimize land‐use change where possible or maintain and improve connectivity	Boncoraglio & Saino 2007; Brumm & Naguib 2009
Invasive species	competition in the acoustic landscape resulting in signal masking	native species producing vocalizations	prevent future invasions and manage current invasive species	Grant & Grant 2010; Farina et al. 2013
**Translocations**				
Small founder populations	formation of cultural bottlenecks, accelerated by serial translocations	populations/species with multiple vocalization types	increase founding population size where possible select representative sample of vocal diversity when choosing birds to translocate	Parker et al. 2012; Valderrama et al. 2012, 2013
Translocation of juveniles	withdrawal of learning resulting in rapid vocal divergence	juvenile birds	use adult birds (or birds with crystallized song) in translocations	Potvin & Clegg 2015
Founding populations from multiple sources	reduced success of territory formation and maintenance assortative mating in translocated populations	birds with vocal dialects (mainly vocal learners, as described above)	increase founding population size to counteract increased mate selectiveness use individuals from the same source where possible select source populations with similar dialects to reduce variation in founding population monitor populations over multiple generations to determine the full extent of assortative mating following the translocation explore the potential of familiarizing birds with other dialects pre‐release	Rowe & Bell 2007; Bradley et al. 2013, 2014; Valderrama et al. 2013
**Ex situ conservation**				
Small, isolated populations	cultural drift may be accelerated in small, isolated populations	vocal learners particular concern for species with complex songs	manage species in larger groups where possible, or retain greater levels of connectivity by moving birds between populations more frequently	Laiolo & Tella 2007; Purushotham & Robin 2016
Differences between wild and ex situ habitats	acoustic adaptation to ex situ habitats different acoustic landscapes due to non‐native species assemblages	species producing vocalizations	reduce number of generations birds are kept in captivity increase similarity between in situ and ex situ environments (e.g., denseness of planting, reduce anthropogenic noise, and reproduce native species composition)	Potvin & Clegg 2015
Adaptation to captivity	release from selection pressure resulting in large scale vocal divergence from wild‐type	species held in captivity for many generations	reduce number of generations birds are kept in captivity minimize differences between in situ and ex situ habitats breeding management to ensure that individual ancestors do not become over‐represented	Honda & Okanoya 1999; Tanimoto et al. 2017
**Reintroductions**				
Ex situ breeding	discussed above	discussed above	discussed above	discussed above
Release from ex situ populations	vocalizations poorly adapted to new environments	species producing vocalizations particular concern for closed‐ended learners	reduce differences between in situ and ex situ environment reduce number of generations in captivity increase founding population size to counteract increased mate selectiveness select source populations with similar dialects to each other or to in situ population to reduce variation in newly formed populations explore potential of familiarizing birds with other dialects pre‐release	Rowe & Bell 2007; Bradley et al. 2013, 2014; Valderrama et al. 2013
	presence of divergent vocal dialects (between wild and released birds or between different populations of released birds)	species with vocal dialects		

### Monitoring

The conspicuous nature of vocalizations means they are easy to measure, even in complex environments, making them a useful noninvasive tool for monitoring (Teixeira et al. 2019). With recent advances in recording technologies and analysis, such as autonomous recording units, it has become possible to collect large amounts of acoustic data with comparatively little effort through passive acoustic monitoring (PAM) (Brandes 2008). Bioacoustic methods perform as well as traditional point counts in a number of cases (Alquezar & Machado 2015; Darras et al. 2018). There remains a need to create standardized practices for acoustic detection surveys (Darras et al. 2018), but a wealth of data with conservation relevance can be collected using these methods.

Acoustic monitoring can provide useful baseline data by examining the spatial and temporal variation of sound (e.g., Pieretti et al. 2011; Rodriguez et al. 2014; Sebastian et al. 2016). Vocalizations extracted from recordings can be used for a number of purposes, including detecting species presence or absence, identifying preferred habitats, detecting juveniles, and determining predator abundance from alarm calls (Teixeira et al. 2019).

Interindividual variation within species allows conservation practitioners to improve the resolution of baseline data (Terry et al. 2005). The ability to discriminate between vocalizations of different individuals can be useful for population censuses (Terry et al. 2005). However, this requires that all birds in a given area vocalize during the sampling period or that researchers have some knowledge of the proportion of birds singing (e.g., if males sing and females do not). If not all birds vocalize, or if vocalizations are biased toward certain demographics or areas (e.g., Legare et al. 1999), population sizes may be underestimated or habitat use may be misinterpreted. The ability to identify individuals by assigning vocalizations to known birds is considerably harder but, when possible, provides useful, high‐resolution data. For example, this can allow researchers to assess how life‐history traits, such as survival, vary among individuals (Terry et al. 2005).

Bioacoustic data can be used to measure responses to environmental perturbations or human disturbance. Anthropogenic noise can affect birds in a number of ways, including altering habitat use and influencing the characteristics of vocal signals (Ortega 2012). Deichmann et al. (2017) used PAM to examine the impacts of natural gas exploration on avian biodiversity and found diversity increases as distance from the drilling site increases. Such information can be used to minimize the impacts of future disturbances and advise conservation programs. Acoustic monitoring can also be employed to evaluate the success of conservation interventions, providing useful evidence for future efforts. Buxton and Jones (2012) used acoustic monitoring to confirm breeding and document population increases of seabirds after the eradication of introduced Arctic foxes (*Alopex lagopus*) in the Aleutian Archipelago. Similarly, individual identification could be useful in postrelease monitoring to determine the fate of specific individuals.

### Artificial Playback for Conspecific Attraction

The presence of conspecifics can attract birds to a habitat, but natural conspecific attraction can be unreliable when conspecifics are rare or absent in new habitats (Crates et al. 2017). Artificial playback of vocalizations can be used in place of conspecifics to reinforce existing populations or encourage animals to colonize new areas (Reed & Dobson 1993). This technique has been used to increase local populations of a number of species (e.g., Ward & Schlossberg 2004; Hahn & Silverman 2007), but is not always successful. Bayard and Elphick (2012) found no evidence of a response to broadcast in Saltmarsh Sparrows (*Ammodramus caudacutus*), possibly due to insufficient cues, already saturated habitats, or broadcast in unsuitable areas.

Conspecific attraction could be used to create communities by attracting multiple species simultaneously to a single site (e.g., DeJong et al. 2015). This would be especially useful for colonizing newly restored habitats or replenishing protected areas. However, past attempts highlight the need to consider the effects of community change on both target and nontarget species. DeJong et al. (2015) found that populations of focal species increased near playback speakers, but populations of some nonfocal species declined and suggest that differences in response between species could relate to interspecific competition.

Although artificial playback for conspecific attraction is a simple and cost‐effective method, we believe that current evidence shows a need for understanding species’ biology and ecology to accurately predict outcomes. Where communities contain multiple endangered species, it is essential to consider the risks associated with conspecific attraction—an increase of one species of conservation concern could lead to the decline of another.

### In Situ Conservation

Large‐ and small‐scale changes in land use can isolate previously contiguous populations, promoting song divergence through drift in the separate populations and through adaptation to changed habitat structure (both for improved acoustic transmission and due to changes in morphological features). Closed‐ended learners may be particularly vulnerable to habitat change because they would be unable to alter their song to transmit well in the new environment. We suggest that preventing land‐use change and protecting species as contiguous populations in the same habitat would help prevent vocal change. Where this is not possible, maintaining or improving connectivity between populations may reduce divergence in song characteristics.

Although limiting the impact of invasive species is a key goal of many conservation efforts, the impact of invasive species on vocalizations is rarely considered. When new species enter the acoustic landscape, resident species may alter song characteristics (Grant & Grant 2010) or become masked by the new vocalizations. Acoustic monitoring of Mediterranean shrubland revealed that the invasive Red‐billed Leiothrix (*Leiothrix lutea*) is acoustically dominant in the landscape, competing with and potentially lowering the density of native species (Farina et al. 2013). If native species cannot compete with invaders in the acoustic landscape, they may not be able to adequately communicate and breed. Preventing future invasions is important to maintain acoustic landscapes for conservation of native species.

### Translocations

Translocations often involve small founder populations, so the formation of cultural bottlenecks is a concern. Serial translocations, where populations from successful translocations are used as source populations for future translocations, may increase the rate of song differentiation among populations, resulting in population divergence, isolation, and reduced retention of animals near release sites. In the North Island Kokako, in translocated populations, songs are shorter and of higher frequency and phrase repertoires are lower than in source populations (Valderrama et al. 2012, 2013). Vocal activity is also markedly reduced, potentially reducing immigration and retention of birds in already small populations (Valderrama et al. 2012). When examining serial translocations in North Island Saddlebacks, Parker et al. (2012) reported reduced song type sharing between translocated and ancestral populations in successive interventions (9.8% shared after the first translocation, 9.2% after the second, and 3.3% after the third). Withdrawal of learning could also cause rapid divergence of songs in recently translocated populations. Moving adult birds with crystallized songs would be preferable to moving juveniles when aiming to limit changes in vocalizations. The withdrawal of learning effect is poorly understood in many species, so monitoring vocalizations after interventions would provide vital information for future conservation programs.

To improve genetic diversity during interventions, multiple source populations may be used for translocations. However, if source populations have different dialects, this may affect territory formation and mate choice. In the North Island Kokako, which is the subject of intensive conservation management, local songs elicit stronger responses from territory‐holding pairs than foreign songs (Bradley et al. 2013), although this result is not consistent among sites (Valderrama et al. 2013). Further examination of responses to dialects across populations is necessary to determine the nuances of differential discrimination. Populations (or individuals) that respond similarly to local and foreign dialects would more easily integrate into new mixed‐dialect populations, making them potential targets for conservation interventions. However, choosing birds based on response may result in inadvertent selection for response and associated traits. Similarly, assortative mating with respect to dialect is common in Kokako; across 10 multisource translocations over 18 years (1993–2011), Kokako mated assortatively in most seasons at all 5 sites. Very few mixed‐dialect pairs formed (Bradley et al. 2014), and mixed‐dialect pairs took considerably longer to form than matched pairs (Rowe & Bell 2007). The long‐term impact of mate selection based on dialect is not clear. First‐generation Kokako hatched at translocation sites do not appear to show preferences for their fathers’ dialects, lending support to theories on familiarity. However, sample sizes are too small to draw clear conclusions. Two first‐generation females paired, one with a male of dialect similar to her father's and the other with a male of a different dialect (Rowe & Bell 2007). Monitoring of mate choice in populations over multiple generations would help determine the overall impact of selection based on dialect over time.

Where dialects are a conservation concern, several mitigation methods could be used: increase starting population size to counteract increased selectiveness; use individuals from the same source population where possible; and select populations with similar dialects to reduce dialect differences in the new group. It is possible that familiarizing birds (either adults or juveniles) with different dialects using playback before translocations could alter preferences and reduce selectiveness in mate choice. MacDougall‐Shackleton et al. (2001) report that preference for natal‐dialect song attenuated in birds exposed to foreign‐dialect song when they were 1 year old. More research is required into the feasibility of this technique. However, if familiarity with dialects reduces aversion to foreign‐dialect mates, conservation practitioners may be able to familiarize birds with all dialects in their new population before the interventions take place, thus improving mating success.

### Ex Situ Conservation

Populations in ex situ management are often small and isolated, which may accelerate the rate of cultural drift (Laiolo & Tella 2007). Housing species in larger groups ex situ where possible or retaining greater levels of group connectivity, for example, by moving birds between populations more frequently, could help to combat the effects of drift. Reducing the number of generations birds are held in ex situ populations would also reduce the likelihood of building up large song differences. These mitigations are particularly important for species with complex songs because these may undergo accelerated rates of drift compared with simple songs (Purushotham & Robin 2016).

Breeding birds ex situ may promote song divergence from the wild‐type through acoustic adaptation, meaning birds will be poorly adapted when reintroduced. Although captive environments are necessarily different from those in situ, matching the environments as closely as possible would limit song evolution. Small changes could be made to increase similarities, such as matching the denseness of vegetation in wild habitats and reducing the presence of anthropogenic noise, such as air‐conditioning units. Again, reducing the number of generations birds spend in unfamiliar environments during conservation interventions may also reduce adaptation because acoustic adaptation can increase with time (Potvin & Clegg 2015).

Captive environments may also differ from those in situ due to species compositions. Mixed‐species enclosures often hold a range of species that would not overlap in the wild, creating an unnatural acoustic landscape. As with invasive species, birds may alter their vocalizations, but if signals become masked, then breeding success in these aviaries may be reduced. Although there is currently little research in this area, potential issues could be avoided by housing species in natural assemblages from the same geographic location.

In captive populations, breeding pairs are usually assigned rather than allowed to form naturally to maximize genetic variability. However, animals limited in mate choice often show reduced reproductive success (Martin & Shepherdson 2012). We hypothesize that a lack of acoustic separation from males with high‐quality or local‐dialect songs may alter females’ perceived mate availability, resulting in females reducing reproductive efforts with their assigned mates. Allowing mate choice or providing appropriate acoustic separation could help alleviate this problem and increase breeding success.

Although reducing the number of generations in captivity could act to reduce divergence in vocal communication, this may not always be possible, and some birds have already been conserved ex situ for many generations (e.g., the ‘Alalā [*Corvus hawaiiensis*] [Tanimoto et al. 2017]).

Prolonged captive breeding could result in species becoming adapted to captivity. Surprisingly, very few studies examine song differences between wild and captive individuals. Tanimoto et al. (2017) compared vocalizations of the ‘Alalā between past wild and current captive populations, finding similar numbers of call types, but significantly different repertoires. Because dialects change over time, it is not possible to say how much of a role captivity played in these changes. All ‘Alalā were brought into captivity, so it is not possible to conduct a contemporary comparison.

The song of the domestic Bengalese Finch (*Lonchura striata domestica*) is more syntactically complex than that of its wild counterpart, the White‐rumped Munia (*L. striata*) (Honda & Okanoya 1999). Several possible explanations for these differences have been proposed, including the lack of predation pressure in captivity for Bengalese Finches (Honda & Okanoya 1999); need for species identification by White‐rumped Munia in mixed‐species flocks (Kagawa et al. 2012); lower levels of corticosterone in Bengalese Finches (Suzuki et al. 2012); and selective breeding for traits correlated with song complexity, such as reproductive output and parental care, in Bengalese Finches (Suzuki et al. 2013). Domestication exerts far stronger selection pressures on populations than captive breeding. However, this example highlights some potential mechanisms of change during long‐term ex situ management.

Many of the situations that give rise to song divergence, such as release from predation and reduced stress, are unavoidable in captivity. However, the steps outlined above, such as minimizing differences in environment and enabling mate choice, may slow or reduce overall change. Breeding management strategies are already commonplace in zoological collections for maintaining genetic and demographic viability (Ballou et al. 2010). These same techniques, such as ensuring individual lineages do not become overrepresented in the breeding pool, may also limit song changes during captive breeding.

### Reintroductions

Many issues facing reintroduction efforts stem from breeding ex situ. The negative effects of adaptation to captivity are highlighted by the relative success rates of translocations and reintroductions. Fischer and Lindenmayer (2000) report that 31% of translocations of wild animals have been successful, but only 13% of translocations with captive animals have been successful. Birds with vocalizations adapted to captive environments may signal less efficiently on release to the wild (Tanimoto et al. 2017). We expect this would be more detrimental to closed‐ended learners, which would not be able to alter songs for their new environment.

Divergence from wild‐type vocalizations may cause problems similar to those seen in translocations with multiple sources if reintroductions aim to supplement preexisting populations, reintroductions use multiple source populations, or multiple reintroductions to a single site are planned. If dialects have diverged, all of these scenarios may affect territory formation or result in increased mate selectiveness and possibly reduced gene flow. Although dialect‐based assortative mating between wild and captive birds has not been studied, it may be expected based on previous studies of female preference (e.g., Searcy et al. 2002) and evidence from translocations (Rowe & Bell 2007; Bradley et al. 2014). We speculate that if reintroduced birds are unable to breed with wild populations or to persist as stable populations themselves and if reintroduced birds compete with native populations, then competition created by reintroductions may hasten rather than prevent extinctions.

Improving the success of reintroductions will involve steps in captivity to reduce song divergence, as outlined above (reduce habitat differences and reduce number of generations in captivity). Recommendations for reintroductions are similar to those for translocations: increase the size of release groups, choose birds with similar dialects to each other and to the source population where possible, and use adult birds to prevent rapid change after release. Moreover, exposing birds both in situ and ex situ to the dialects of other populations may improve integration and mating success, although further research is needed to determine the feasibility of this approach.

## Conclusion

Considerable progress has been made in the use of vocalizations to aid conservation, particularly with bioacoustics used to monitor populations and survey biodiversity. Although research into acoustic monitoring continues to grow, research examining the adverse effects of variation in vocalizations during conservation programs is lacking. There are a number of important questions that should be addressed in order to build a better evidence base for conservation practitioners. First, little is known about the actual effect of dialects on mating. Although females often show preference for local songs, this may not reflect how they mate in conservation settings. Although some evidence for assortative mating in the wild exists (e.g., the Kokako), evidence from mixed‐dialect populations suggests that dialect‐based assortative mating may be due only to familiarity. If this is true, it may be possible to mitigate the problem by familiarizing young birds with the dialects they may encounter during conservation efforts. Similarly, additional research is needed into the long‐term effects of dialect‐based assortative mating; assortative mating may not be maintained over multiple generations. Long‐term population monitoring after intervention would be necessary to determine this. Furthermore, understanding of the interplay between dialects and gene flow remains limited. Individuals are often closely monitored during conservation to examine breeding success. Thus, it may be possible to determine how dialects affect gene flow by constructing pedigrees within populations. If gene flow persists during interventions in spite of dialects, this knowledge would be extremely beneficial to practitioners. Finally, the evolution of birdsong during ex situ management is poorly understood. Understanding the drivers and extent of acoustic change during conservation breeding is essential for planning of breeding programs and reintroductions. Future research should focus on these knowledge gaps to help practitioners and scientists properly plan for and mitigate potential adverse effects of variation in vocalizations during conservation.

## References

[cobi13465-bib-0001] Alquezar RD , Machado RB . 2015. Comparisons between autonomous acoustic recordings and avian point counts in open woodland savanna. Wilson Journal of Ornithology 127:712–723.

[cobi13465-bib-0002] Angeloni L , Schlaepfer MA , Lawler JJ , Crooks KR . 2008. A reassessment of the interface between conservation and behaviour. Animal Behaviour 75:731–737.

[cobi13465-bib-0003] Baker MC . 1996. Depauperate meme pool of vocal signals in an island population of singing honeyeaters. Animal Behaviour 51:853–858.

[cobi13465-bib-0004] Baker MC , Mewaldt LR . 1978. Song dialects as barriers to dispersal in white‐crowned sparrows, *Zonotrichia leucophrys nuttalli* . Evolution 32:712–722. 28567938 10.1111/j.1558-5646.1978.tb04624.x

[cobi13465-bib-0005] Baker MC , Thompson DB , Sherman GL , Cunningham MA , Tomback DF . 1982. Allozyme frequency in a linear series of song dialect populations. Evolution 36:1020–1029. 28567817 10.1111/j.1558-5646.1982.tb05470.x

[cobi13465-bib-0006] Ballentine B , Hyman J , Nowicki S . 2004. Vocal performance influences female response to male bird song: an experimental test. Behavioral Ecology 15:163–168.

[cobi13465-bib-0007] Ballou JD , Lees C , Faust LJ , Long S , Lynch C , Bingaman Lackey L , Foose TJ . 2010. Demographic and genetic management of captive populations. Pages 219–252 in Kleiman, DG , Thompson, KV , Baer, CK , editors. Wild mammals in captivity: principles and techniques for zoo management. University of Chicago Press, Chicago, Illinois.

[cobi13465-bib-0008] Bayard TS , Elphick CS . 2012. Testing for conspecific attraction in an obligate saltmarsh bird: can behavior be used to aid marsh restoration? Wetlands 32:521–529.

[cobi13465-bib-0009] Beecher MD , Brenowitz EA . 2005. Functional aspects of song learning in songbirds. Trends in Ecology and Evolution 20:143–149. 16701358 10.1016/j.tree.2005.01.004

[cobi13465-bib-0010] Berger‐Tal O , Blumstein DT , Carroll S , Fisher RN , Mesnick SL , Owen MA , Saltz D , St. Claire CC , Swaisgood RR . 2016. A systematic survey of the integration of animal behavior into conservation. Conservation Biology 30:744–753. 26548454 10.1111/cobi.12654

[cobi13465-bib-0011] Bessa‐Gomes C , Danek‐Gontard M , Cassey P , Moller AP , Legendre S , Clobert J . 2003. Mating behaviour influences extinction risk: insights from demographic modelling and comparative analysis of avian extinction risk. Annales Zoologici Fennici 40:231–245.

[cobi13465-bib-0012] BirdLife International . 2018. State of the world's birds.

[cobi13465-bib-0013] Boncoraglio G , Saino N . 2007. Habitat structure and the evolution of bird song: a meta‐analysis of the evidence for the acoustic adaptation hypothesis. Functional Ecology 21:134–142.

[cobi13465-bib-0104] Bluff LA , Kacelnik, A , Rutz C . 2010. Vocal culture in New Caledonian crows Corvus moneduloides. Biological Journal of the Linnean Society 101:767–776.

[cobi13465-bib-0014] Bradley DW , Molles LE , Waas JR . 2013. Local‐foreign dialect discrimination and responses to mixed‐dialect duets in the North Island kōkako. Behavioral Ecology 24:570–578.

[cobi13465-bib-0015] Bradley DW , Molles LE , Waas JR . 2014. Post‐translocation assortative pairing and social implications for the conservation of an endangered songbird. Animal Conservation 17:197–203.

[cobi13465-bib-0016] Brandes TS . 2008. Automated sound recording and analysis techniques for bird surveys and conservation. Bird Conservation International 18:S163–S173.

[cobi13465-bib-0017] Brumm H , Naguib M . 2009. Environmental acoustics and the evolution of bird song. Advances in the Study of Behavior 40:1–33.

[cobi13465-bib-0018] Buxton RT , Jones IL . 2012. Measuring nocturnal seabird activity and status using acoustic recording devices: applications for island restoration. Journal of Field Ornithology 83:47–60.

[cobi13465-bib-0019] Byers BE , Kroodsma DE . 2009. Female mate choice and songbird song repertoires. Animal Behaviour 77:13–22.

[cobi13465-bib-0020] Cade TJ , Jones CG . 1993. Progress in restoration of the Mauritius kestrel. Conservation Biology 7:169–175.

[cobi13465-bib-0021] Catchpole CK , Slater PJB . 2008. Bird song: biological themes and variation. 2nd edition. Cambridge University Press, Cambridge, United Kingdom.

[cobi13465-bib-0022] Chesser RK , Rhodes OE , Sugg DW , Schnabel A . 1993. Effective sizes for subdivided populations. Genetics 135:1221–1232. 8307332 10.1093/genetics/135.4.1221 PMC1205752

[cobi13465-bib-0023] Chilton G , Lein MR , Baptista LF . 1990. Mate choice by female white‐crowned sparrows in a mixed‐dialect population. Behavioral Ecology and Sociobiology 27:223–227.

[cobi13465-bib-0024] Chilton G , Lein MR , Dechesne S , Esser C , Cash K , Chilton L , Johnson LS , Walker R . 1996. Songs and sexual responses of female white‐crowned sparrows from a mixed‐dialect population. Behaviour 133:173–198.

[cobi13465-bib-0025] Crates R , Rayner L , Stojanovic D , Webb M , Heinsohn R . 2017. Undetected Allee effects in Australia's threatened birds: implications for conservation conservation. Emu 117:1–15.

[cobi13465-bib-0026] Daniels SJ , Walters JR . 2000. Inbreeding depression and its effects on natal dispersal in red‐cockaded woodpeckers. Condor 102:482–491.

[cobi13465-bib-0027] Darras K , Batáry P , Furnas B , Celis‐Murillo A , Van Wilgenburg SL , Mulyani YA , Tscharntke T . 2018. Comparing the sampling performance of sound recorders versus point counts in bird surveys: a meta‐analysis. Journal of Applied Ecology 55:2575–2586.

[cobi13465-bib-0028] Deichmann JL , Hernández‐Serna A , Delgado CJA , Campos‐Cerqueira M , Aide TM . 2017. Soundscape analysis and acoustic monitoring document impacts of natural gas exploration on biodiversity in a tropical forest. Ecological Indicators 74:39–48.

[cobi13465-bib-0029] DeJong LN , Cowell SD , Nguyen TNN , Proppe DS . 2015. Attracting songbirds with conspecific playback: a community approach. Behavioral Ecology 26:1379–1388.

[cobi13465-bib-0030] Derryberry EP , Seddon N , Derryberry GE , Claramunt S , Seeholzer GF , Brumfield RT , Tobias JA . 2018. Ecological drivers of song evolution in birds: disentangling the effects of habitat and morphology. Ecology and Evolution 8:1–16. 10.1002/ece3.3760 PMC5792612 29435262

[cobi13465-bib-0031] Farina A , Pieretti N , Morganti N . 2013. Acoustic patterns of an invasive species: the Red‐billed Leiothrix (*Leiothrix lutea* Scopoli 1786) in a Mediterranean shrubland. Bioacoustics 22:175–194.

[cobi13465-bib-0032] Ficken RW , Ficken MS , Hailman JP . 1974. Temporal pattern shifts to avoid acoustic interference in singing birds. Science 183:762–763. 17790627 10.1126/science.183.4126.762

[cobi13465-bib-0033] Fischer J , Lindenmayer DB . 2000. An assessment of the published results of animal relocations. Biological Conservation 96:1–11.

[cobi13465-bib-0034] García NC , Tubaro PL . 2018. Dissecting the roles of body size and beak morphology in song evolution in the “blue” cardinalids (Passeriformes: Cardinalidae). Auk 135:262–275.

[cobi13465-bib-0035] Gil D , Gahr M . 2002. The honesty of bird song: multiple constraints for multiple traits. Trends in Ecology and Evolution 17:133–141.

[cobi13465-bib-0036] Grant BR , Grant PR . 1996. Cultural inheritance of song and its role in the evolution of Darwin's finches. Evolution 50:2471–2487. 28565664 10.1111/j.1558-5646.1996.tb03633.x

[cobi13465-bib-0037] Grant BR , Grant PR . 2002. Simulating secondary contact in allopatric speciation: an empirical test of premating isolation. Biological Journal of the Linnean Society 76:545–556.

[cobi13465-bib-0038] Grant BR , Grant PR . 2010. Songs of Darwin's finches diverge when a new species enters the community. Proceedings of the National Academy of Sciences of the United States of America 107:20156–20163. 21048082 10.1073/pnas.1015115107 PMC2996702

[cobi13465-bib-0039] Hafner DJ , Petersen KE . 1985. Song dialects and gene flow in the white‐crowned sparrow, *Zonotrichia leucophrys nuttalli* . Evolution 39:687–694. 28561976 10.1111/j.1558-5646.1985.tb00405.x

[cobi13465-bib-0040] Hahn BA , Silverman ED . 2007. Managing breeding forest songbirds with conspecific song playbacks. Animal Conservation 10:436–441.

[cobi13465-bib-0041] Hart PJ , Hall R , Ray W , Beck A , Zook J . 2015. Cicadas impact bird communication in a noisy tropical rainforest. Behavioral Ecology 26:839–842. 26023277 10.1093/beheco/arv018 PMC4433330

[cobi13465-bib-0042] Hill SD , Ji W , Parker KA , Amiot C , Wells SJ . 2013. A comparison of vocalisations between mainland tui (*Prosthemadera novaeseelandiae novaeseelandiae*) and Chatham Island tui (*P. n. chathamensis*). New Zealand Journal of Ecology 37:214–223.

[cobi13465-bib-0043] Hinde RA . 1956. The biological significance of territories of birds. Ibis 98:340–369.

[cobi13465-bib-0044] Honda E , Okanoya K . 1999. Acoustical and syntactical comparisons between songs of the white‐backed munia (*Lonchura striata*) and its domesticated strain, the Bengalese finch (*Lonchura striata* var. domestica). Zoological Science 16:319–326.

[cobi13465-bib-0045] Irwin DE , Bensch S , Price TD . 2001. Speciation in a ring. Nature 409:333–337. 11201740 10.1038/35053059

[cobi13465-bib-0046] Jiang Y , Bolnick DI , Kirkpatrick M . 2013. Assortative mating in animals. American Naturalist 181:E125–E138. 10.1086/670160 23669548

[cobi13465-bib-0047] Kagawa H , Yamada H , Lin R , Mizuta T , Hasegawa T , Okanoya K . 2012. Ecological correlates of song complexity in white‐rumped munias: the implication of relaxation of selection as a cause for signal variation in birdsong. Interaction Studies 13:263–284.

[cobi13465-bib-0048] Keller LF , Waller DM . 2002. Inbreeding effects in wild populations. Trends in Ecology & Evolution 17:230–241.

[cobi13465-bib-0049] Kirkpatrick M . 2000. Reinforcement and divergence under assortative mating. Proceedings of the Royal Society B: Biological Sciences 267:1649–1655. 10.1098/rspb.2000.1191 PMC1690725 11467428

[cobi13465-bib-0050] Krebs JR , Kroodsma DE . 1980. Repertoires and geographical variation in bird song. Advances in the Study of Behavior 11:143–177.

[cobi13465-bib-0051] Kroodsma D . 2017. Birdsong performance studies: a contrary view. Animal Behaviour 125:e1–e16.

[cobi13465-bib-0052] Laiolo P , Tella JL . 2007. Erosion of animal cultures in fragmented landscapes. Frontiers in Ecology and the Environment 5:68–72.

[cobi13465-bib-0053] Lang AL , Barlow JC . 1997. Cultural evolution in the Eurasian tree sparrow: divergence between introduced and ancestral populations. Condor 99:413–423.

[cobi13465-bib-0054] Leader N , Geffen E , Mokady O , Yom‐Tov Y . 2008. Song dialects do not restrict gene flow in an urban population of the orange‐tufted sunbird, *Nectarinia osea* . Behavioral Ecology and Sociobiology 62:1299–1305.

[cobi13465-bib-0055] Legare ML , Eddleman WR , Buckley PA , Kelly C . 1999. The effectiveness of tape playback in estimating black rail density. Journal of Wildlife Management 63:116–125.

[cobi13465-bib-0056] Lovette IJ , Fitzpatrick JW . 2016. Handbook of bird biology. 3rd edition. Wiley‐Blackwell, New Jersey.

[cobi13465-bib-0057] Luther D . 2009. The influence of the acoustic community on songs of birds in a neotropical rain forest. Behavioral Ecology 20:864–871.

[cobi13465-bib-0058] MacDougall‐Shackleton EA , MacDougall‐Shackleton SA . 2001. Cultural and genetic evolution in mountain white‐crowned sparrows: song dialects are associated with population structure. Evolution 55:2568–2575. 11831670 10.1111/j.0014-3820.2001.tb00769.x

[cobi13465-bib-0059] MacDougall‐Shackleton SA , MacDougall‐Shackleton EA , Hahn TP . 2001. Physiological and behavioural responses of female mountain white‐crowned sparrows to natal‐ and foreign‐dialect songs. Canadian Journal of Zoology 79:325–333.

[cobi13465-bib-0060] Martin MS , Shepherdson DJ . 2012. Role of familiarity and preference in reproductive success in ex situ breeding programs. Conservation Biology 26:649–656. 22809353 10.1111/j.1523-1739.2012.01880.x

[cobi13465-bib-0061] Metcalf OC , Ewen JG , McCready M , Williams EM , Rowcliffe JM . 2019. A novel method for using ecoacoustics to monitor translocation behaviour in an endangered passerine. Methods in Ecology and Evolution 10:626–636.

[cobi13465-bib-0062] Miskelly CM , Powlesland RG . 2013. Conservation translocations of New Zealand birds, 1863–2012. Notornis 60:3–28.

[cobi13465-bib-0063] Mundinger PC . 1980. Animal cultures and a general theory of cultural evolution. Ethology and Sociobiology 1:183–223.

[cobi13465-bib-0064] Nowicki S , Searcy WA . 2005. Song and mate choice in birds: how the development of behavior helps us understand function. Auk 122:1–14.

[cobi13465-bib-0065] Ortega CP . 2012. Chapter 2: Effects of noise pollution on birds: a brief review of our knowledge. Ornithological Monographs 74:6–22.

[cobi13465-bib-0066] Parker KA , Anderson MJ , Jenkins PF , Brunton DH . 2012. The effects of translocation‐induced isolation and fragmentation on the cultural evolution of bird song. Ecology Letters 15:778–785. 22590997 10.1111/j.1461-0248.2012.01797.x

[cobi13465-bib-0067] Parker KA , Hauber ME , Brunton DH . 2010. Contemporary cultural evolution of a conspecific recognition signal following serial translocations. Evolution 64:2431–2441. 20394665 10.1111/j.1558-5646.2010.01013.x

[cobi13465-bib-0068] Pearse WD , Morales‐Castilla I , James LS , Farrell M , Boivin F , Davies TJ . 2018. Global macroevolution and macroecology of passerine song. Evolution 72:944–960. 29441527 10.1111/evo.13450

[cobi13465-bib-0069] Petrinovich L , Patterson T , Baptista LF . 1981. Song dialects as barriers to dispersal: a re‐evaluation. Evolution 35:180–188. 28563445 10.1111/j.1558-5646.1981.tb04871.x

[cobi13465-bib-0070] Pieretti N , Farina A , Morri D . 2011. A new methodology to infer the singing activity of an avian community: the Acoustic Complexity Index (ACI). Ecological Indicators 11:868–873.

[cobi13465-bib-0071] Planque R , Slabbekoorn H . 2007. Spectral overlap in songs and temporal avoidance in a Peruvian bird assemblage. Ethology 114:262–271.

[cobi13465-bib-0072] Podos J , Warren PS . 2007. The evolution of geographic variation in birdsong. Advances in the Study of Behavior 37:403–444.

[cobi13465-bib-0073] Poesel A , Fries AC , Miller L , Gibbs HL , Soha JA , Nelson DA . 2017. High levels of gene flow among song dialect populations of the Puget Sound white‐crowned sparrow. Ethology 123:581–592.

[cobi13465-bib-0074] Potvin DA , Clegg SM . 2015. The relative roles of cultural drift and acoustic adaptation in shaping syllable repertoires of island bird populations change with time since colonization. Evolution 69:368–380. 25496402 10.1111/evo.12573

[cobi13465-bib-0075] Potvin DA , Parris KM , Mulder RA . 2013. Limited genetic differentiation between acoustically divergent populations of urban and rural silvereyes (*Zosterops lateralis*). Evolutionary Ecology 27:381–391.

[cobi13465-bib-0076] Purushotham CB , Robin VV . 2016. Sky island bird populations isolated by ancient genetic barriers are characterized by different song traits than those isolated by recent deforestation. Ecology and Evolution 6:7334–7343. 28725401 10.1002/ece3.2475 PMC5513277

[cobi13465-bib-0077] Reed JM , Dobson AP . 1993. Behavioural constraints and conservation biology: conspecific attraction and recruitment. Trends in Ecology and Evolution 8:253–256. 21236161 10.1016/0169-5347(93)90201-Y

[cobi13465-bib-0078] Robin VV , Katti M , Purushotham C , Sancheti A , Sinha A . 2011. Singing in the sky: song variation in an endemic bird on the sky islands of southern India. Animal Behaviour 82:513–520.

[cobi13465-bib-0079] Rodriguez A , Gasc A , Pavoine S , Grandcolas P , Gaucher P , Sueur J . 2014. Temporal and spatial variability of animal sound within a neotropical forest. Ecological Informatics 21:133–143.

[cobi13465-bib-0080] Rowe SJ , Bell BD . 2007. The influence of geographic variation in song dialect on post‐translocation pair formation in North Island kokako (*Callaeas cinerea wilsoni*). Notornis 54:28–37.

[cobi13465-bib-0081] Searcy WA . 1982. The evolutionary effects of mate selection. Annual Review of Ecology and Systematics 13:57–85.

[cobi13465-bib-0082] Searcy WA . 1992. Song repertoire and mate choice in birds. American Zoologist 32:71–80.

[cobi13465-bib-0083] Searcy WA . 1997. The response of male and female song sparrows to geographic variation in song. Condor 99:651–657.

[cobi13465-bib-0084] Searcy WA , Nowicki S , Hughes M , Peters S . 2002. Geographic song discrimination in relation to dispersal distances in song sparrows. American Naturalist 159:221–230. 10.1086/338509 18707375

[cobi13465-bib-0085] Sebastian J , Gasc A , Gaucher P , Aubin T , Réjou‐Méchain M , Sueur J . 2016. Screening large audio datasets to determine the time and space distribution of Screaming Piha birds in a tropical forest. Ecological Informatics 31:91–99.

[cobi13465-bib-0086] Slabbekoorn H , Smith TB . 2002. Bird song, ecology and speciation. Philosophical Transactions of the Royal Society B: Biological Sciences 357:493–503. 10.1098/rstb.2001.1056 PMC1692962 12028787

[cobi13465-bib-0087] Snowberg LK , Benkman CW . 2007. The role of marker traits in the assortative mating within red crossbills, *Loxia curvirostra* complex. Journal of Evolutionary Biology 20:1924–1932. 17714309 10.1111/j.1420-9101.2007.01372.x

[cobi13465-bib-0088] Soha JA , Nelson DA , Parker PG . 2004. Genetic analysis of song dialect populations in Puget Sound white‐crowned sparrows. Behavioral Ecology 15:636–646.

[cobi13465-bib-0089] Soma M , Takahasi M , Hasegawa T , Okanoya K . 2006. Trade‐offs and correlations among multiple song features in the Bengalese Finch. Ornithological Science 5:77–84.

[cobi13465-bib-0090] Sueur J . 2002. Cicada acoustic communication: potential sound partitioning in a multispecies community from Mexico (Hemiptera: Cicadomorpha: Cicadidae). Biological Journal of the Linnean Society 78:379–394.

[cobi13465-bib-0091] Suzuki K , Ikebuchi M , Okanoya K . 2013. The impact of domestication on fearfulness: a comparison of tonic immobility reactions in wild and domesticated finches. Behavioural Processes 100:58–63. 23962671 10.1016/j.beproc.2013.08.004

[cobi13465-bib-0092] Suzuki K , Yamada H , Kobayashi T , Okanoya K . 2012. Decreased fecal corticosterone levels due to domestication: a comparison between the white‐backed Munia (*Lonchura striata*) and its domesticated strain, the Bengalese finch (*Lonchura striata* var. domestica) with a suggestion for complex song evolution. Journal of Experimental Zoology 317:561–570. 22927235 10.1002/jez.1748

[cobi13465-bib-0093] Tanimoto AM , Hart PJ , Pack AA , Switzer R , Banko PC , Ball DL , Sebastián‐González E , Komarczyk L , Warrington MH . 2017. Changes in vocal repertoire of the Hawaiian crow, *Corvus hawaiiensis*, from past wild to current captive populations. Animal Behaviour 123:427–432.

[cobi13465-bib-0094] Teixeira D , Maron M , van Rensburg BJ . 2019. Bioacoustic monitoring of animal vocal behavior for conservation. Conservation Science and Practice 1:e72.

[cobi13465-bib-0095] Terry A , Peake T , McGregor P . 2005. The role of vocal individuality in conservation. Frontiers in Zoology 2:10. 15960848 10.1186/1742-9994-2-10 PMC1183234

[cobi13465-bib-0096] Thielcke G . 1973. On the origin of divergence of learned signals (songs) in isolated populations. Ibis 115:511–516.

[cobi13465-bib-0097] Valderrama SV , Molles LE , Waas JR . 2012. Effects of population size on singing behavior of a rare duetting songbird. Conservation Biology 27:210–218. 22979901 10.1111/j.1523-1739.2012.01917.x

[cobi13465-bib-0098] Valderrama SV , Molles LE , Waas JR , Slabbekoorn H . 2013. Conservation implications of song divergence between source and translocated populations of the North Island Kōkako. Journal of Applied Ecology 50:950–960.

[cobi13465-bib-0099] Verzijden MN , ten Cate C , Servedio MR , Kozak GM , Boughman JW , Svensson EI . 2012. The impact of learning on sexual selection and speciation. Trends in Ecology & Evolution 27:511–519. 22705159 10.1016/j.tree.2012.05.007

[cobi13465-bib-0100] Ward MP , Schlossberg S . 2004. Conspecific attraction and the conservation of territorial songbirds. Conservation Biology 18:519–525.

[cobi13465-bib-0101] Whitehead H , Rendell L . 2015. The cultural lives of whales and dolphins. University of Chicago Press, Chicago, Illinois.

[cobi13465-bib-0102] Wright TF , Dahlin CR , Salinas‐Melgoza A . 2008. Stability and change in vocal dialects of the yellow‐naped amazon. Animal Behaviour 76:1017–1027. 19727330 10.1016/j.anbehav.2008.03.025 PMC2598431

[cobi13465-bib-0103] Zink RM , Barrowclough GF . 1984. Allozymes and song dialects: a reassessment. Evolution 38:444–448. 28555894 10.1111/j.1558-5646.1984.tb00303.x

